# Correlating the Crystal Structure and Optical Response of DNA-Stabilized Ag_16_Cl_2_ Clusters

**DOI:** 10.1021/acs.jpcc.5c02959

**Published:** 2025-08-28

**Authors:** Maya Khatun, Sami Malola, Hannu Häkkinen

**Affiliations:** † Department of Physics, Nanoscience Center, 4168 University of Jyväskylä, FI-40014 Jyväskylä, Finland; ‡ Department of Chemistry, Nanoscience Center, University of Jyväskylä, FI-40014 Jyväskylä, Finland

## Abstract

We investigate the electronic structure and optical response of the DNA-stabilized silver cluster (DNA)_2_-Ag_16_Cl_2_ using density functional theory in its ground-state and linear-response forms. Our calculations are based on experimental crystal data, where DNA strands, mutated by guanosine–inosine change, are protecting an inorganic Ag_16_Cl_2_ core [ Cerretani  Nanoscale Adv. 2022, 4, 3212−3217 36132821 10.1039/d2na00325bPMC9416947]. We find a remarkable sensitivity of the computed optical absorption spectrum on (i) the level of approximations of how the clusters’ environment is modeled (implicit continuum solvent model, with or without the explicit crystal water molecules in the vicinity of the DNA strands), (ii) the level of the approximations regarding the electron–electron exchange-correlation functionals, and (iii) the minute differences in crystal packing in subclusters in the crystal unit cell. Our work highlights the challenges for high-fidelity computational modeling of the electronic structure of these hybrid bionano materials and points to need to further develop computational methods toward efficient sampling of dynamical behavior to understand better the correlations between measured and computed properties.

## Graphical abstract

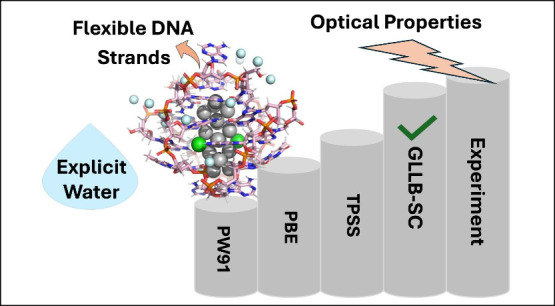

## Introduction

DNA-stabilized silver nanoclusters (DNA-AgNCs) are biocompatible near-infrared (NIR) emitters, making them highly promising for bioimaging and sensing applications.^1–7^ These nanoclusters exhibit great potential for detecting viruses, including severe acute respiratory syndrome coronavirus 2 (SARS-CoV-2).^8,9^ Additionally, researchers find applications in cell recognition, food safety, and the catalytic reduction of highly toxic nitrobenzene compounds.^10–12^

DNA-AgNCs consist of small aggregates of 10 to 30 silver atoms wrapped within DNA strands, with the sequence of the DNA bases determining their structure and photophysical properties.^13–18^ This sequence-structure–property relationship has facilitated the synthesis of an array of atomically precise NIR-emitting DNA-AgNCs.^14^ Recently, these nanoclusters have accumulated special interest due to their exceptional photophysical properties and large Stokes shifts.^15,19,20^ They constitute a new family of fluorescent emitters offering numerous advantages over other emerging metal nanoclusters (MNCs), such as DNA-templated copper nanoclusters. They exhibit a broader and more tunable fluorescence wavelength range, exceptional stability, higher fluorescence quantum yields, and lower toxicity.^21–23^ Notably, DNA-AgNCs have achieved fluorescence quantum yields (QY) as high as 64%.^24^

The generation of DNA-AgNCs on the nucleation domain of the DNA molecule does not noticeably affect the other regions of the DNA,^25^ allowing these parts to fold into their typical shapes and bind with complementary DNA sequences. Therefore, DNA-AgNCs can be combined with various amplification methods to achieve sensitive detection of nucleic acids without labeling.^26,27^ The secondary structures of DNA are crucial for highly emissive DNA-AgNCs, and AgNCs stabilize secondary DNA structures such as hairpin DNA, duplex DNA, and parallel-motif DNA triplexes. It has also been reported that the fluorescence of AgNCs encapsulated within a Hoogsteen triplex DNA structure can be toggled on and off with pH changes.^28^

Despite numerous experimental studies on DNA secondary structures and DNA-AgNCs, theoretical research remained limited for a long time due to the lack of atomic structures obtained from experiments. However, early theoretical studies aided understanding of the interaction between cationic silver or very small silver clusters and DNA bases.^29–32^ These studies highlighted that addition of just one atom to the cluster size can significantly influence interactions with DNA.^32^ Other research indicates that hydrogen bonds are crucial in determining the structure of solvated silver-mediated cytosine tetramers through novel interplanar interactions, opening the possibility of designing three-dimensional metal-mediated duplexes using interplanar hydrogen bonds as fundamental building blocks.^33^

Recently, significant progress has been made with the reporting of crystal structures for double-stranded DNA-encapsulated silver clusters, Ag_8_ and Ag_16_.^3,34,35^ The Ag_16_ cluster wrapped by two 10-base DNA strands (5′-CAC­CTA­GCGA-3′) is notable for its NIR-emitting fluorescent properties, rod-like and slightly chiral geometry, and substantial Stokes shift.^13,36,37^ However, a previous study had mis-assigned two sites outside the Ag_16_ core as partially occupied silver atoms. Subsequent reanalysis using complementary techniques, including electrospray ionization mass spectrometry, NMR, DFT calculations, and refined single-crystal X-ray analysis, corrected this error by identifying these sites as chlorides.^36^ This breakthrough facilitated the first theoretical studies on this system using density functional theory (DFT).^36,38–42^

Cerretani et al. have synthesized a mutated Ag_16_Cl_2_ cluster where guanosines (G) at positions 7 and 9 (G79) were replaced by inosines (I79), yielding the sequence 5′-CAC­CTA­ICIA-3′.^15^ Otherwise, the overall structure is very similar to the G79 form.^36^ The I79 mutant has been shown to exhibit a higher quantum yield and distinct fluorescent properties compared to the G79 cluster, but the effects induced by mutations have not been thoroughly studied. Interestingly, the crystal structure^15^ of the I79 mutant contains six slightly different subclusters in the crystal unit cell, giving a possibility to make density-functional theory (DFT) calculations for each of them separately.

In this work, we investigate the electronic structure of the I79 variant of the Ag_16_Cl_2_ cluster using the experimental crystal data^15^ as the starting point. We benchmark several exchange-correlation functionals, examine the effect of explicit solvent interactions and explore how subtle variations in DNA strand orientation influence the electronic and optical properties of the system. To achieve a comprehensive understanding, we analyze key spectral features, including absorption peaks, the density of states (DOS), molecular orbitals (MOs), and circular dichroism (CD), providing deeper insights into the optical properties and environmental effect within the cluster. While being a follow-up study to our previous research on the electronic structure of the G79 form of the Ag_16_Cl_2_ cluster,^38^ this work yields significant new information on the sensitivity of the electronic structure of the DNA-AgNCs on details of the DNA layer and the environment, which has not been stressed in previous studies.

## Computational Methods

The model structures for I79 clusters were prepared for the computational study by considering the crystal structure (PDB accession code: 7XLW)^15^ and adding hydrogen atoms according to the valency of atoms. The crystal unit cell contained six clusters, from which one was excluded from computations since its DNA layer was not fully resolved. The remaining five subclusters (labeled **A**, **B**, **D**–**F**, see Figure 1) were subjected to ground-state and linear-response DFT studies that were conducted by using the GPAW software.^43,44^ The initial optimization of the added hydrogen atoms was performed using the PBE^45^ density functional and using a real-space grid spacing of 0.25 Å. After the optimization, various density functional approximations (DFAs) (GLLB-SC,^46^ TPSS,^46,47^ PBE,^45^ PW91 ^48^) were considered to converge the Kohn–Sham electronic ground state, and grid spacing of 0.30 Å was used. These grid spacings were selected to achieve a balance between computational accuracy and efficiency. A vacuum layer of 6 Å was included around the system in all cases. The PAW atomic setups for the Ag atom include scalar-relativistic effects.

**Figure 1:**
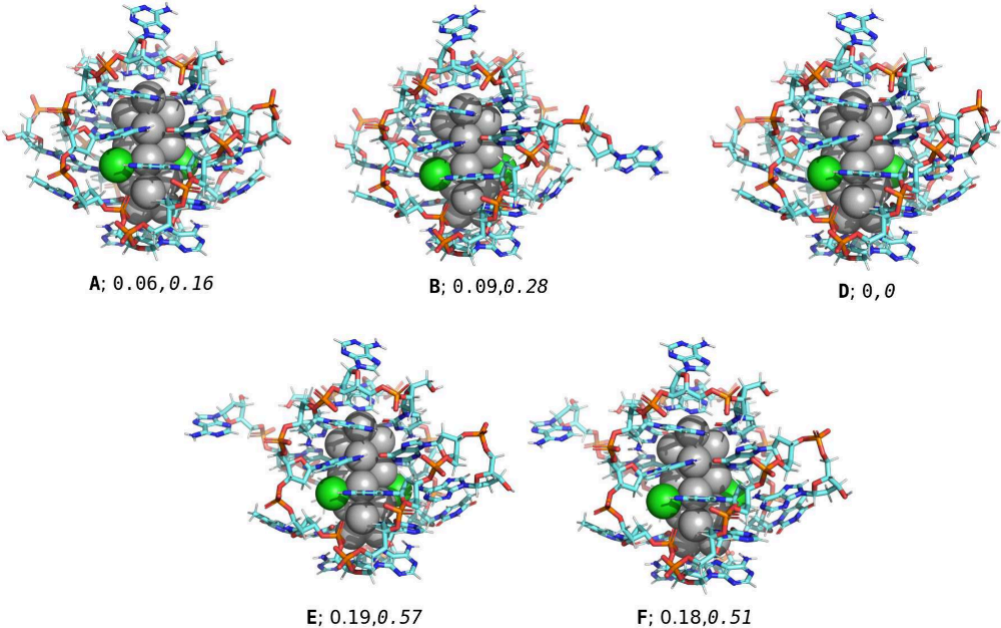
Visualization of the five subclusters of the inosine double mutant I79 from the experimental crystal structure data (PDB accession code: 7XLW)^15^ (subcluster C was not fully resolved and is omitted in this work). The numbers denote RMSD values (Ag_16_Cl_2_, *DNA*; in Å) with respect to the subcluster D. The visualization was done using PyMOL; Ag: gray (spheres), Cl: green (spheres), C: aquamarine (sticks), N: blue (sticks), P: orange (sticks), O: red (sticks), H: white (sticks).

In all calculations, the overall charge of the cluster was set to −12|e| for reasons that are discussed in the Results section. Previously, we have documented the crucial effect of incorporating solvation effects in the DFT calculations of the highly negatively charged G79 cluster.^38^ Here, in addition to employing an implicit continuum solvent model with parameters for water adopted from the work by Held and Walter et al.,^49^ we performed a reference set of calculations incorporating also the explicit water molecules in the vicinity of DNA around each considered subcluster, as found in the experimental structure data.

To analyze the ground-state electronic structure, Kohn–Sham electronic states were decomposed into contributions from different atoms by projecting the density of states (PDOS) onto spherical harmonics within defined spherical volumes around each atom.^50^ Summation over all angular momenta and specified atoms was performed to determine the final localization weights. The cutoff radii for the elements were as follows: Ag: 1.5 Å, Cl: 1.3 Å, P: 0.8 Å, O: 0.7 Å, C: 0.7 Å, N: 0.7 Å, H: 0.5 Å.

Optical absorption and circular dichroism (CD) spectra were calculated starting from the ground-state wave functions and using the linear-response time-dependent density functional theory (LR-TDDFT) as installed in GPAW,^51^ with the PBE functional used as the TDDFT kernel. All these calculations were performed using an implicit continuum solvent model, additionally, in some calculations the water molecules found in the crystal structure close to the subcluster were included as well, akin to the ground-state calculations described above. We will note these conditions while discussing the results.

Individual oscillator strengths (for absorption) and rotatory strengths (for CD) were smoothed using 0.1 eV Gaussians and summed to produce continuous spectra. Peaks in the optical absorption spectra were analyzed using dipole transition contribution maps (DTCM) calculated from time-dependent density functional perturbation theory (TD-DFPT).^52^ DTCM illustrates the constructive and screening contributions to the total dipole transition moment at selected energies in the Kohn–Sham (ground-state) electron–hole basis. These maps were calculated as averages over the three Cartesian coordinate directions of the electric field polarization. The polarization directions were aligned with the system’s principal axes of inertia. Local charges of atoms and atomic groups were analyzed using the Bader charge approch.^53^ Frontier molecular orbitals were visualized using Jmol,^54^ and geometries were visualized using PyMOL software.^55^

## Results and Discussion

Our work builds from the previous findings based on the G79 cluster by Malola et al., where silver core exhibited a charge distribution corresponding to the presence of six “free” electrons, which originate from charge balance between the phosphate groups, silver atoms, and chloride ions.^36,38^ It is also reported that the silver atoms interacted strongly with guanine nucleobases, particularly at the N7 and N1 positions of G7 and G9. Notably, in the G79 cluster, G9 was deprotonated at the N1 position, a process influenced by silver ions.^38^ In the present work on inosine double mutant, similar interactions are expected at the modified I7 and I9 sites. The I9^–^ modification, being negatively charged like G9^–^, plays a crucial role in modulating charge distribution and the overall stability of the system.

In this study, we first evaluated various methods to determine the most suitable approach for analyzing the inosine double mutant, I79 ([(DNA)_2_-Ag_16_Cl_2_]^12–^, 5′-CAC­CTA­ICIA-3′), which has a total charge of – 12 |e|. This charge corresponds to the expected configuration of six “free” electrons like G79 in the silver core, calculated from 18 negatively charged phosphate groups, 16 electrons donated by silver atoms, two electron-withdrawing chloride ions, and two negatively charged I9^–^. To address the concern regarding the high negative charge (−12 |e|) of the I79 system, we employed an implicit continuum solvent model in GPAW, which accounts for dielectric screening effects in aqueous environments.^49^ This treatment stabilizes the charged species and mitigates artificial distortions in the electronic structure, particularly in the frontier orbital energies. The use of highly charged states in DNA-stabilized silver clusters is consistent with prior work in the field^38^ on G79 system where the electronic structure was analyzed across various charge states. The findings showed that without implicit solvent, phosphate contributions introduced partially occupied states near the Fermi level, which influenced low-lying optical transitions. When including implicit solvation, these phosphate states were stabilized (shifted to lower energy), and opened a clear HOMO–LUMO gap using the charge state consistent with the experimental observations. Consequently, the computed absorption spectrum of the cluster with implicit solvent matched experimental data nearly precisely. This demonstrates that high-charge states can be physically meaningful and computationally stable in the presence of solvent screening. We have followed the same strategy in our present study.

The six-electron species holds critical importance in the study of DNA-AgNCs due to its unique role in determining their electronic and photophysical properties. ESI-MS analyses revealed that DNA-AgNCs are partially oxidized, resulting in an effective valence electron count (N_0_) that is lower than the total number of silver atoms (N) in the core. Among these, clusters with N_0_ = 6 exhibit nanosecond-lived fluorescence and wavelength scaling properties consistent with their rod-like geometries.^36^ This specific electron configuration not only governs excited-state dynamics but also serves as a key benchmark for electronic structure calculations. Notably, DFT-calculated optical absorbance for the 6-electron system aligned well with experimental results, further validating the theoretical approach.^38^ Additionally, DFT result explained the two distinct resonances observed in the ^35^Cl-nuclear magnetic resonance (NMR) spectrum and revealed solvent-accessible “channels” that may facilitate halide exchange.

### Performance of the Exchange-Correlation Functional

We considered one of the six subclusters (**A**, Figures 1) and calculated optical absorption spectra with four different DFAs in absence and presence of explicit water molecules and compared with the experimental spectrum. The ground state Kohn–Sham wave functions were calculated using the PW91, PBE, TPSS, and GLLB-SC functionals, followed by LR-TDDFT calculations using the PBE functional with an implicit solvent model (Figure 2).

**Figure 2:**
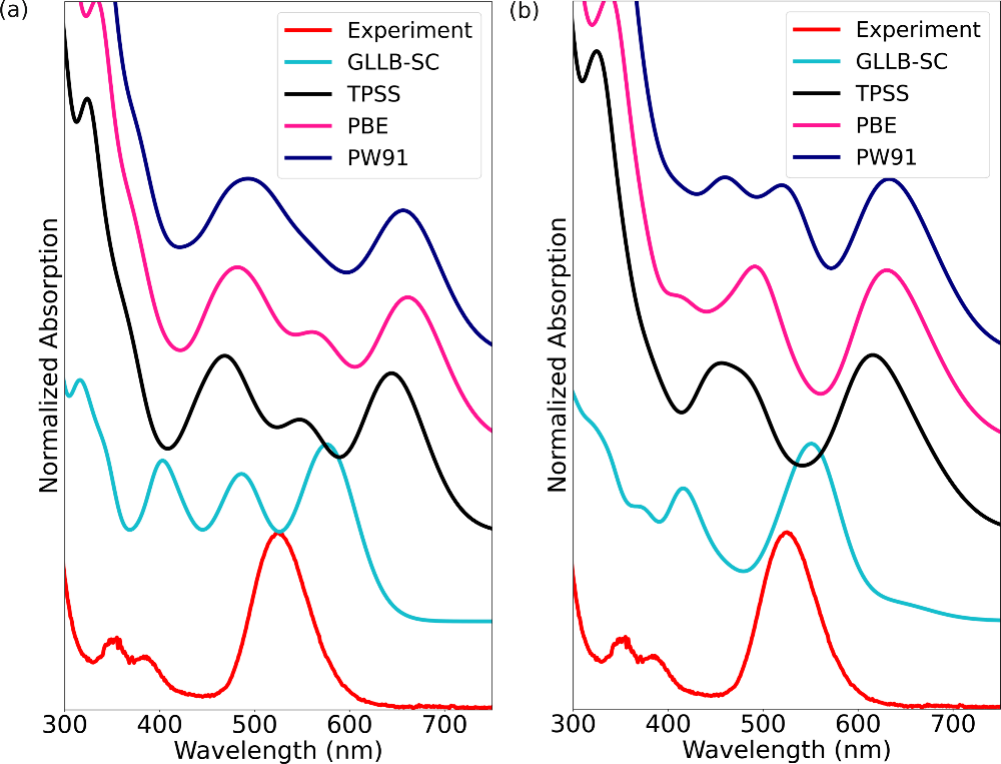
Calculated optical absorption spectra of I79 subcluster **A** in (a) absence and (b) presence of explicit water molecules compared to experimental^15^ spectrum. In both cases, ground-state Kohn–Sham wave functions were calculated using the PW91, PBE, TPSS, and GLLB-SC functionals. LR-TDDFT calculations used the PBE functional as the kernel. Implicit solvent model was applied.

The experimental spectrum has one strong absorption peak at 525 nm and two weak peaks at 383 and 355 nm in a very similar fashion as the corresponding G79 variant that we have studied before.^36,38^ We note that from all eight calculated spectra in Figure 2, the one calculated with the GLLB-SC functional for the ground-state wave functions and including the water molecules from the crystal structure yields on overall best agreement with the experiment. Its general form includes a strong absorption at 551 nm and two weaker ones at 416 and 365 nm. Interestingly, using only the implicit solvent to describe the aqueous environment in the calculation has a quite dramatic effect, yielding three quite equal absorptions in the range of 400–600 nm. We also note that all GGA-derived functionals (PBE, PW91, and TPSS) perform all quite the same way in both sets of calculations to model the aqueous environment, yielding similar spectral shapes and peak positions. However, all spectra are strongly red-shifted with respect to the experiment.

### Geometrical Configuration of Sub-Clusters and Effect on Optical Spectra

As mentioned above, the I79 crystal structure contains six subclusters in the unit cell and five are resolved to atomic accuracy in the DNA layer so that they can be considered in the DFT calculations (Figure 1). Although these clusters are generally similar, they exhibit differences in the orientation of DNA bases relative to the silver core and variations in the number of water molecules found around them in the crystal structure. Specifically, the orientation of the adenine at position 10 (A10) of the second strand varies among the configurations. Subclusters **A** and **D** have A10 oriented toward the silver-chloride core. In contrast, **B**, **E**, and **F** have A10 oriented outward from the silver-chloride core. These and other detailed differences are reflected in the root-mean-square deviation (RMSD) values when the clusters are aligned on top of each other (Figure 1). We note that the RMSD for the inorganic core stays very small but it is appreciably higher for the DNA layer, reflecting also the visual impression in Figure 1.

Based on the DFA analysis discussed above, we selected the GLLB-SC functional for the Kohn–Sham wave functions. Figure 3a shows that when the environment is described only by the implicit solvent, subcluster **D** shows a spectrum that closely matches the experimental data, both in a general shape and for location of the strong absorption peak (518 nm vs 525 nm for calculated and measured peak, respectively). When the water molecules are included in the calculation (Figure 3b), the first peak blue-shifts closer to the experimental peak but develops a shoulder on the high-energy side. Under these conditions, subcluster **A** has a spectrum in a slightly better agreement with the experiment. We note that the minute differences in the atomic structure between the subcluster yield a surprisingly strong effect on their optical absorption spectra, both in the shape and location of the peaks. To understand this effect better, we next analyze a subset of the data in more detail. We selected three systems: **A** and **D** with the implicit solvent only to describe the environment, and **A** also including the water molecules.

**Figure 3:**
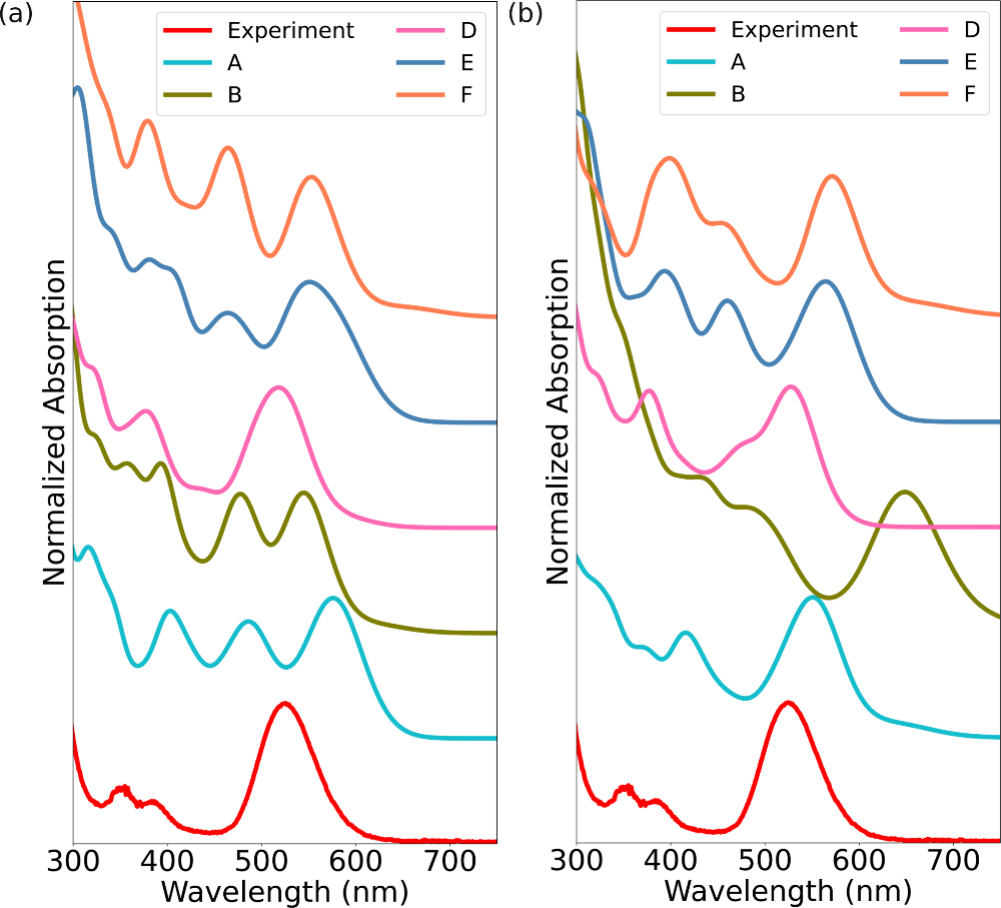
Calculated optical absorption spectra of I79 subclusters **A, B, D–F** in (a) absence and (b) presence of explicit water molecules compared to the experimental^15^ spectrum. In both cases, ground-state Kohn–Sham wave functions were calculated using the GLLB-SC functional. LR-TDDFT calculations used the PBE functional as the kernel. Implicit solvent model was applied.

### Electronic Structure

#### HOMO–LUMO Energy Gaps

Figure 4 displays the computed absorption spectra for the three selected systems discussed above, as well as the density of electron states as projected to the natural components of the clusters: Ag, Cl, base, sugar, phosphate, and explicit water. Comparing Figures 4a and 4b one sees that the magnitude of the energy gap between the highest occupied molecular orbital (HOMO) and the lowest unoccupied molecular orbital (LUMO) correlates well with the computed optical gap. **D** has the largest HOMO–LUMO gap (HLG= 1.908 eV) and the largest optical gap, followed by **A** including the water molecules (HLG = 1.755 eV) and **A** with implicit solvent (HLG = 1.719 eV).

**Figure 4:**
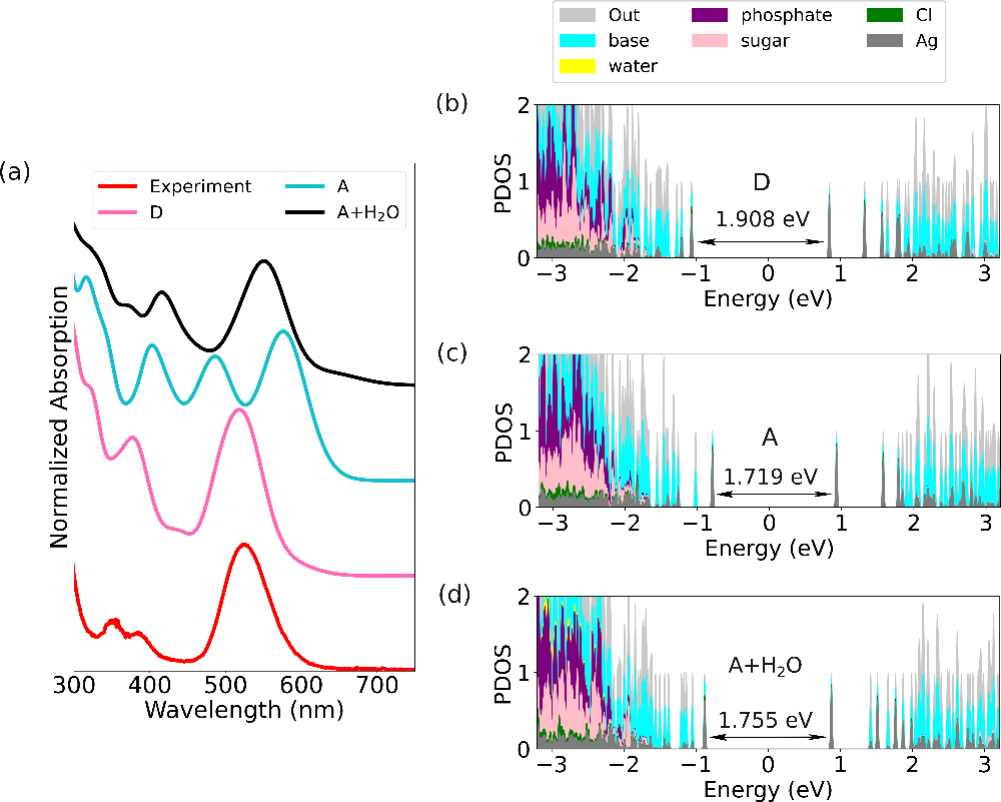
(a) Calculated optical absorption spectra of I79 subclusters **D** and **A** (implicit solvent) and **A** including the water molecules, as compared to experimental spectrum (red). In all cases, ground-state Kohn–Sham wave functions were calculated using the GLLB-SC functional. (b–d) Projected density of electronic states (PDOS) to the specified atom groups. “Out” denotes weights from the spatial distribution of electrons that are not captured by the analysis. Values of the HOMO–LUMO energy gaps shown as well.

Figures 4b and 5 show that in all cases, the HOMO and LUMO are localized on the silver atoms, which agrees with our previous findings for the G79 variant.^36,38^ Notably, in subcluster **D**, the HOMO is also contributed by I9^–^ (Figure 5). The density of states for energies just below the HOMO does not vary significantly from one system to another. Interestingly, there is a change in energy-ordering of some of the lowest-energy unoccupied orbitals: for **D** and **A** with implicit solvent, the LUMO and LUMO+1 orbitals are similar between the systems, but for **A** with the explicit water molecules included there is a new orbital LUMO+1 showing up while LUMO+2 is similar to LUMO+1 in the other two cases. We interpret this change in energy-ordering to be caused by direct interactions between water molecules and DNA bases, which cannot be captured by the implict solvent model.

**Figure 5:**
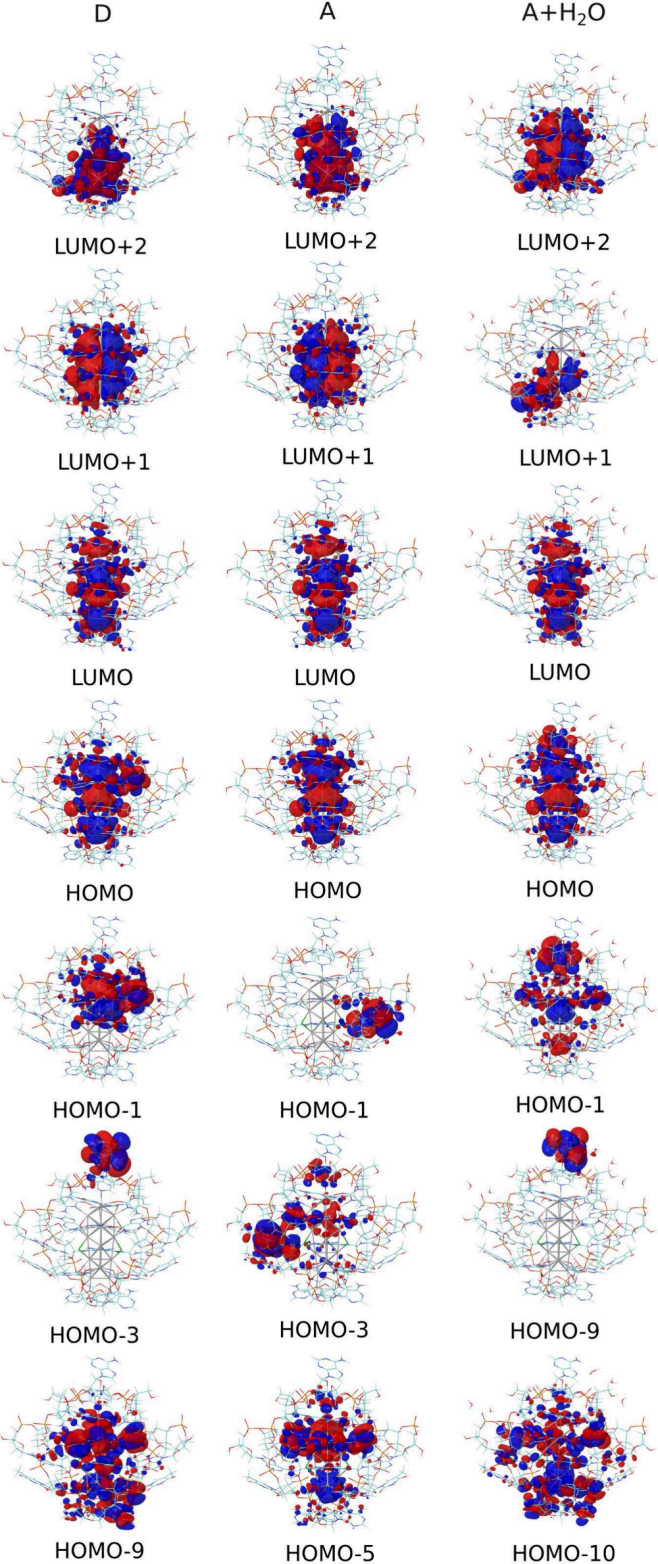
Frontier molecular orbitals (FMOs) of the same systems and conditions as shown in Figure 4.

One such case is analyzed in Figure 6 for subcluster **A**. Water molecules are positioned near the DNA bases, particularly between the silver atoms and DNA bases or phosphate groups. Figure 6 highlights hydrogen bonding interactions between water molecules and DNA nucleobases. The measured hydrogen bond distances are as follows: Ag–N (I7 and I9) 2.2–2.6 Å, N–H_2_O 3.0–3.8 Å, O (I7 and I9)-H_2_O 3.4–4.6 Å, Ag–H_2_O 4.2 Å, P–H_2_O 3.3 Å, and H_2_O–H_2_O 2.7, 2.8, and 4.9 Å. The zoomed-in view emphasizes these distances (marked in yellow) between the water molecules and DNA nucleobases, specifically showing interactions with the nitrogen atoms of C3 and C4, as well as the oxygen of I9. These hydrogen bonding interactions contribute to the overall rigidity and stability of the molecular configuration.

**Figure 6:**
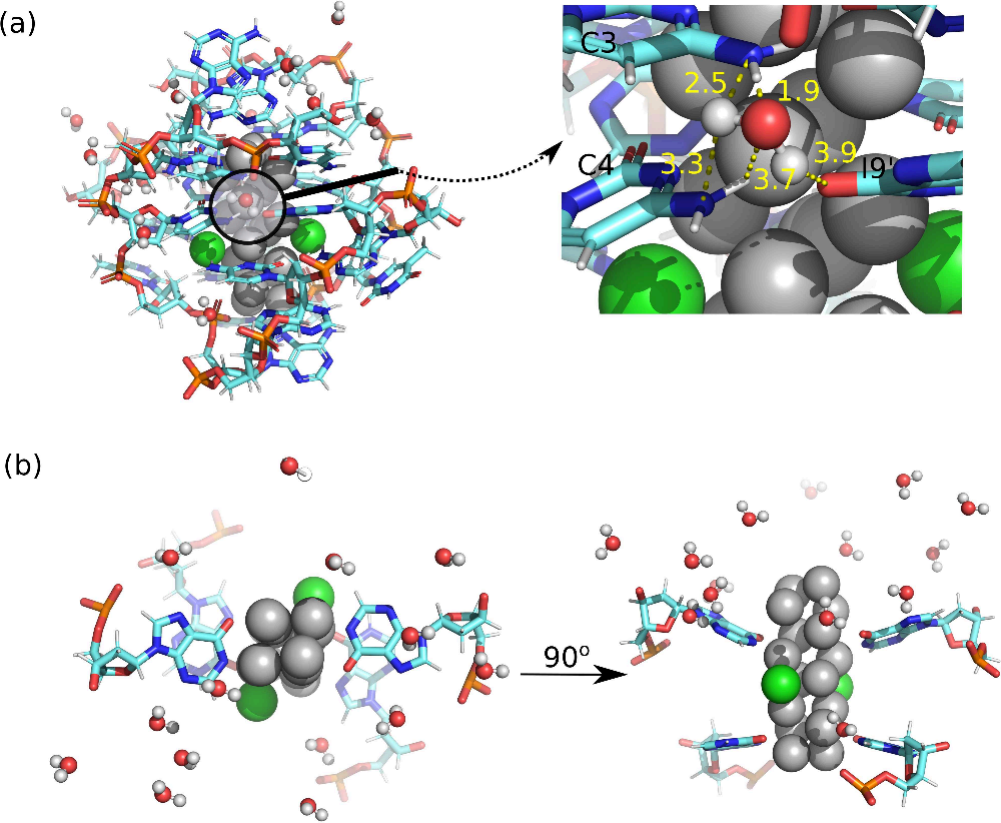
Visualization of I79 subcluster **A** including the water molecules found in the experimental crystal structure. (a) shows the overall strucure in left and the zoomed-in section on the right highlights the distances (yellow, Å) between water molecule and DNA nucleobases, specifically showing the nitrogen atoms of C3 and C4, as well as the oxygen of I9. (b) Side and top views of I7 and I9 in both strands of I79 (5′-CAC­CTA­ICIA-3′) are presented for structural clarity.

We also note here case-specific variations in frontier molecular orbitals among the subclusters. In **D**, HOMO–1 is primarily contributed by silver and I9^–^, while in **A**, it is localized at A10, **A** with explicit water localized at silver, A2, and I9^–^. HOMO–9 of **A** with explicit water and HOMO–3 of **D** are contributed by A2′, where water molecules inducing energy shifts in the **A** subunit. Explicit water molecules of **A** directly contribute to HOMO–9 and HOMO–1, inducing an effect at I7 of LUMO+1, which in turn causes **D** to exhibit LUMO+3, resembling LUMO+1 of **A** in the presence of explicit water molecules. Furthermore, in **A**, HOMO–3 is largely localized on I9^–^, with partial contributions from the silver core. Lower-energy MOs exhibit I7, I9^–^, and silver-centered localization, with some examples including HOMO–9 (**D**), HOMO–5 (**A**), and HOMO–10 (**A** in explicit water).

Many of the frontier orbitals display particle-in-a-box characteristics in the silver core. HOMO and LUMO exhibit two and three nodal planes, respectively, oriented perpendicular to the longitudinal direction. In contrast, LUMO+1 and LUMO+2 display nodal planes in the transverse direction. LUMO+1 (**A** in explicit water) differs from **D** and **A**, as it includes significant contributions from I7. The symmetry considerations imply a strong dipole-allowed HOMO–LUMO longitudinal transition.

#### Atomic Charges

Bader charge analysis of subcluster **D**, **A**, and **A+H**_**2**_O of I79 shows a slight positive charge for silver atoms, with values of 0.367, 0.362, and 0.372 e, respectively, closely aligning with the previously reported G79 system (0.369 e, Table S1). Chloride maintains a negative charge in all subclusters **D** (−0.607 e), **A** (−0.647 e), and **A+H**_**2**_O (−0.630 e), which is consistent as well with G79 (−0.653 e). Interestingly, we do find differences in charges calculated per sugar, base or phosphate. Sugars are less positively charged for **D** (1.536 e), **A** (1.436 e), and **A+H**_**2**_O (1.541 e) as compared to G79 (1.618 e). Phosphates and bases are negatively charged for all the cases. Charge per base varies for different subclusters **D** (−0.432 e), **A** (−0.412 e), **A+H**_**2**_O (−0.452 e), with a slightly higher value for G79 (−0.465 e). Similarly, the charge per phosphates varies for different subcluster **D** (−2.152 e), **A** (−2.055 e), **A+H**_**2**_O (−2.137 e), with G79 exhibiting a slightly larger charge of −2.209 e. The presence of water molecules slightly enhances the negative charge of negatively charged groups while reducing the positive charge of positively charged groups, suggesting that environmental conditions influence partial charge distribution.

#### Analysis of Absorption Peaks

The absorption peaks were analyzed using Dipole Transition Contribution Maps (DTCM), considering three specific cases: subclusters **D**, **A**, and **A** in explicit water molecules. As shown in Figure 7, for all three cases, the lowest energy absorption peaks arises from silver-to-silver transitions, corresponding to HOMO to LUMO transitions predominantly localized on the silver core (Figure 5).

**Figure 7:**
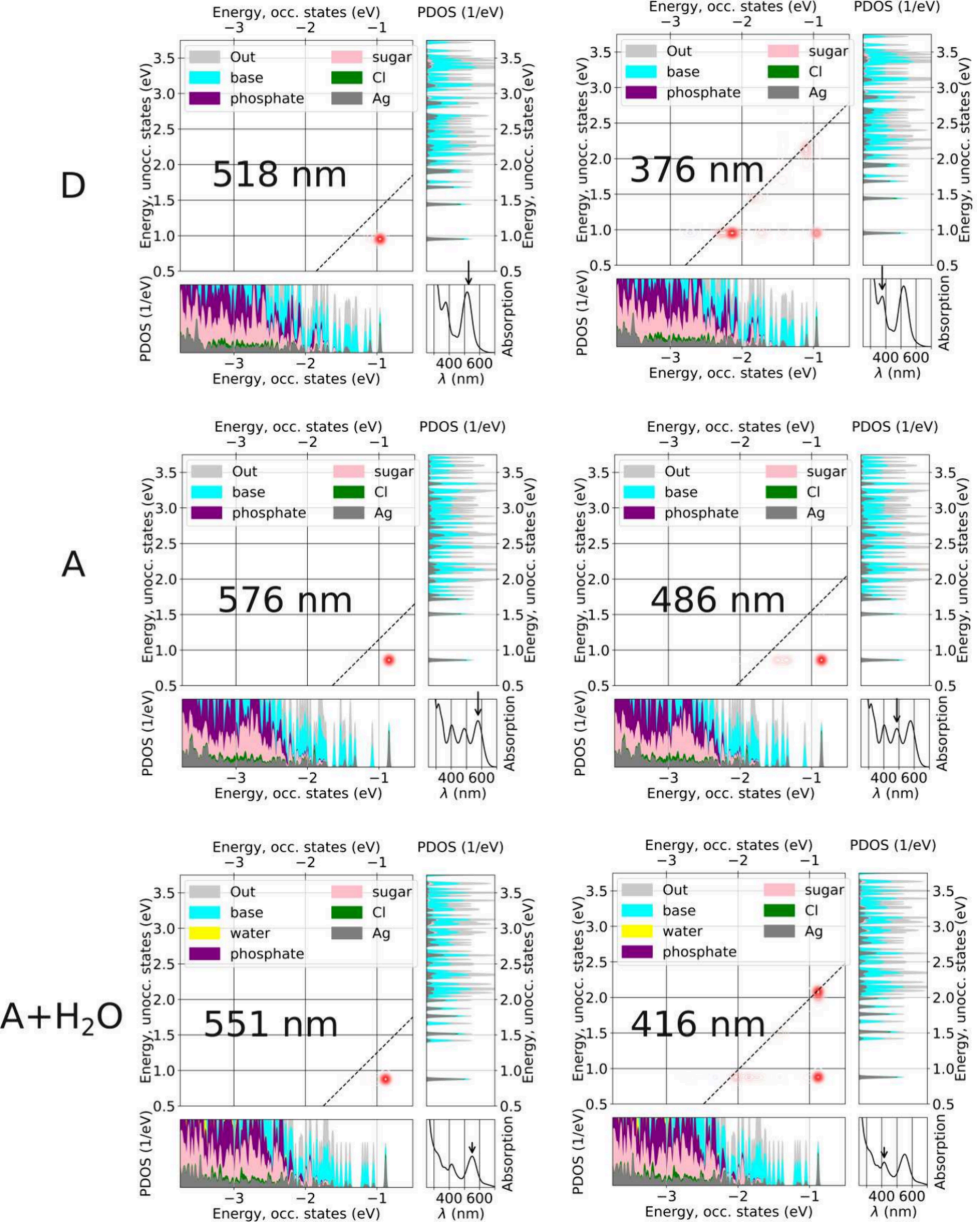
Dipole Transition Contribution Map (DTCM) analysis for the same systems and conditions as in Figure 4. In each collage of panels, the bottom right corner reproduces the calculated absorption spectrum and the arrow indicates the analyzed peak whose position is also given in nm. The correlation plot shows the contributions to the total transition dipole moment of the given peak by single-electron transitions from occupied Kohn–Sham orbitals (bottom left panel) to the empty orbitals (right vertical panel). The Kohn–Sham orbitals are projected onto the natural components of the system as in Figure 4.

The second absorption peak presents more complex transitions. In **D** and **A** with explicit water molecules, this peak results from a combination of the HOMO-to-LUMO, base-to-LUMO, and HOMO-to-base transitions. However, in **A** computed with the implicit solvent, the second peak (486 nm) mirrors the first (576 nm) but appears at higher energy (shorter wavelength), indicating a splitting effect due to a HOMO–LUMO transition shift. The higher-energy peaks exhibit combined transitions involving silver-to-silver, silver-to-base, and base-to-silver contributions (Figure S1). Additionally, a fourth peak at 316 nm () originates from base-to-base, base-to-silver, and phosphate-to-silver transitions. It is interesting to note that despite the transition energy, the HOMO–LUMO transition has always a visible contribution in the DTCM analysis.

### Circular Dichroism Spectra

DNA-AgNCs clusters are fundamentally interesting hybrid nanosystems for studying the chiral response. Chirality can be present in many levels from the inner metal core to the outermost DNA strands. In this section we analyze the chiroptical properties of I79 clusters. The results of previous sections have underlined the sensitivity of the optical properties of these clusters to small differences in the structure and in the solvent environment. To make the comparison between experiments and calculations easier we report here the calculated CD spectra of the three representative subclusters with respect to the position of the first absorption peak. In practice, it means that we have applied a subcluster specific shift to each CD spectrum to align the position of the lowest-energy calculated linear absorption peak with the experimental value of 525 nm (Figure 4a).

The aligned computational CD spectra are shown in Figure 8. We can see that all subclusters **A**, **D**, and **A** with explicit water show qualitatively similar CD spectra. All three subclusters have a negative peak at the position of the first linear absorption peak (at 525 nm). This kind of signal has been observed experimentally in many of the DNA-AgNCs clusters with two DNA-strands, especially in 6 electron Ag_15–17_-(DNA)_2_ clusters measured without Cl-ligands. Adding Cl-ligands has been shown to suppress the measured CD signal at longer wavelengths.^13^ The first negative peak is caused by the transitions between the metal core states as indicated already by the DTCM analysis of the first absorption peak (see Figure 7). This means that the metal core and the transitions between the particle-in-a-box like states have chiral activity reflecting the arrangement of the surrounding bases and the DNA strands. At the shorter wavelengths (320–450 nm) there is a wider positive band in the spectrum consisting of 2–3 local maxima. Subclusters **D** and **A** with explicit water have consistent CD spectra. The positive band is located at the position of the two weaker linear absorption peaks and the corresponding wavelength range has transition contributions mainly from the silver core and from the bases. Around 300 nm all of the spectra have a sharp minimum. The overall behavior of the calculated CD spectra coincide nicely with the measured spectra of similar clusters also at shorter wavelengths.^13^

**Figure 8:**
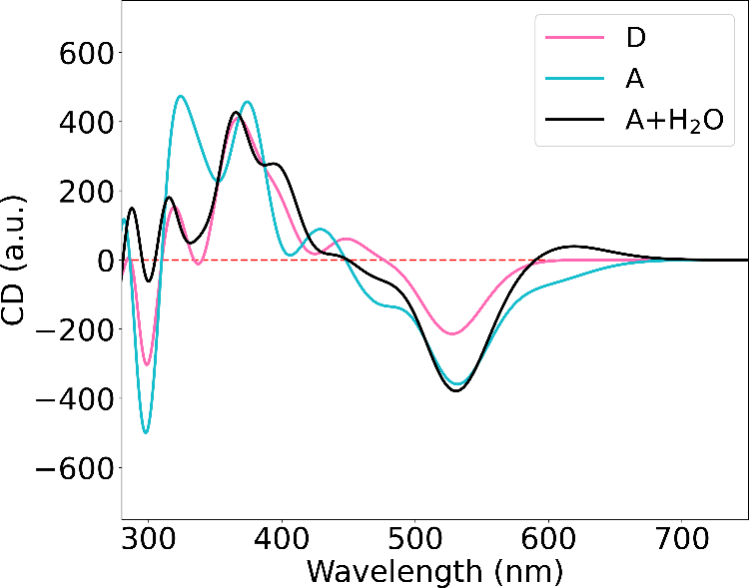
Calculated circular dichroism (CD) spectra for the same systems and conditions as shown in Figure 4.

To summarize, the variations in the calculated CD spectra between subclusters reflect the differences in the exact structures of the clusters and the effects of the water solvent, but as such do not affect the assignment of the cluster handedness. Subclusters **D** and **A** with explicit water are expected to give the best prediction for the CD spectrum, similarly as for the absorption spectra. Because of the missing dynamics and statistical sampling over structure fluctuations, our modeling cannot explain all the effects that are seen in the experiments. Naturally, the peaks of the CD spectrum would in general get smoothened due to the statistical sampling of the dynamical cluster structures. Moreover, the intensity of individual peaks could vary nonuniformly as a function of dynamical effects if the chiral signal in some specific part of the cluster is more vulnerable to even small distortions in the cluster structure. This may be the case for the lowest-energy CD signals originating from the particle-in-a-box metal–metal transitions.

## Conclusions

Our work highlights the necessity to carefully assess several critical assumptions when density functional theory calculations are performed to investigate the ground-state electronic structure and optical response of DNA-stabilized silver nanoclusters, that are interesting hybrid materials exhibiting delocalized electronic structure in the metal core hybridized with the localized molecular states of DNA. Benchmarking against experimental data for optical absorption, we find that the GLLB-SC exchange-correlation functional, in general, performs better than the family of GGA-derived functionals. This may be because the GLLB-SC contains a mechanism for corrections for self-interaction errors and has originally been developed for semiconductor materials where transitions across the band gap involve charge transfer inside the system.

We also found that inclusion of the explicit structure of water molecules in the vicinity of the DNA as found in the crystal structure may improve the description of the electronic structure. This is likely an indirect and local effect, where the strong dipole from the nearby water molecule may shift given localized orbitals in the DNA strands.

The most surprising result is the sensitivity of the computed spectra on apparently small structural variations in the cluster, as exemplified by the five studied subclusters packed in the crystal unit cell. This fact raises caution against interpretation of measured ensemble properties by calculations based on any single structure (whether from crystal data or from computational optimization). DNA itself is known to be a highly dynamical molecule in water at room temperature, and many computational studies on dynamical properties of small isolated metal clusters in gas phase have pointed out the “fluxionality” of their atomic dynamics. It is to be seen to what degree the DNA-stabilized silver clusters show dynamical fluctuations of their atomic structure, and how large a challenge those fluctuations will present when, e.g., circular dichroism spectra should be reliably computed gathering enough fluctuations to yield sufficient statistical sampling.

## Supplementary Material





## Notes

Additional computational data of dipole transition contribution map of higher energy peaks (Figures S1 and S2) of subcluster **A** and partial charge distribution of different atomic or molecular groups at Table S1 (PDF)
Optimized coordinates of the five subclusters, both with and without explicit water molecules (ZIP)
