# Supplementary material for: Correlating the Crystal Structure and Optical Response of DNA-Stabilized Ag16Cl2 Clusters
Source: J Phys Chem C Nanomater Interfaces. 2025 Aug 28;129(36):16155–63. doi: 10.1021/acs.jpcc.5c02959 (PMC12604614; doi:10.1021/acs.jpcc.5c02959)
Supplement: Supplementary file 1 [file jp5c02959_si_001.pdf]

Supporting Information: Correlating the Crystal Structure and Optical Response of DNA-Stabilized $Ag_{16}Cl_2$ Clusters

Maya Khatun,[†] Sami Malola,[†] and Hannu Häkkinen[*,†,‡]

†*Department of Physics, Nanoscience Center, University of Jyväskylä, FI-40014 Jyväskylä, Finland*

‡*Department of Chemistry, Nanoscience Center, University ofJyväskylä, FI-40014 Jyväskylä, Finland*

E-mail: hannu.j.hakkinen@jyu.fi

Contents

List of Tables **S2**

List of Figures **S2**

References **S4**

List of Tables

S1 Charge distribution among different atomic/molecular groups in sub-cluster **D**, **A**, **A+H_2O** of I79 and G79. . . . . S4

List of Figures

S1 The Dipole Transition Contribution Map (DTCM) illustrates the higher energy absorption peak of sub-cluster **A** at 402 nm (indicated by the black arrow) in the absorption spectrum, computed using the GLLB-SC ground state with implicit solvent. The red spots in the top left panel highlight the combined transitions from silver-to-silver (HOMO-to-LUMO), silver-to-base, and base-to-silver. The bottom left panel depicts the projected densities of occupied states (PDOS), while the top right panel displays unoccupied states. S5

S2 The Dipole Transition Contribution Map (DTCM) illustrates the higher energy absorption peak of sub-cluster **A** at 316 nm (indicated by the black arrow) in the absorption spectrum, computed using the GLLB-SC ground state with implicit solvent. The red spots in the top left panel highlight the collective transitions from silver-to-silver (HOMO-to-LUMO), silver-to-base, base-to-silver, base-to-base, and sugar-phosphate-to-silver. The bottom left panel depicts the projected densities of occupied states (PDOS), while the top right panel displays unoccupied states. S6

Table S1: Charge distribution among different atomic/molecular groups in sub-cluster **D**, **A**, **A+H_2O** of I79 and G79.

Atom/Molecule Group	Number (N)	Q/N_D	Q/N_A	Q/N_{A+H_2O}	Q/N_{G79} [S1]
Ag	16	0.367	0.362	0.372	0.369
Cl	2	-0.607	-0.647	-0.630	-0.653
Sugars	20	1.536	1.436	1.541	1.618
Phosphates	18	-2.152	-2.055	-2.137	-2.209
Bases	20	-0.432	-0.412	-0.452	-0.465

References

(S1) Malola, S.; Matus, M. F.; Häkkinen, H. Theoretical Analysis of the Electronic Structure and Optical Properties of DNA-Stabilized Silver Cluster Ag16Cl2 in Aqueous Solvent. *J. Phys. Chem. C* **2023**, *127*, 16553–16559.

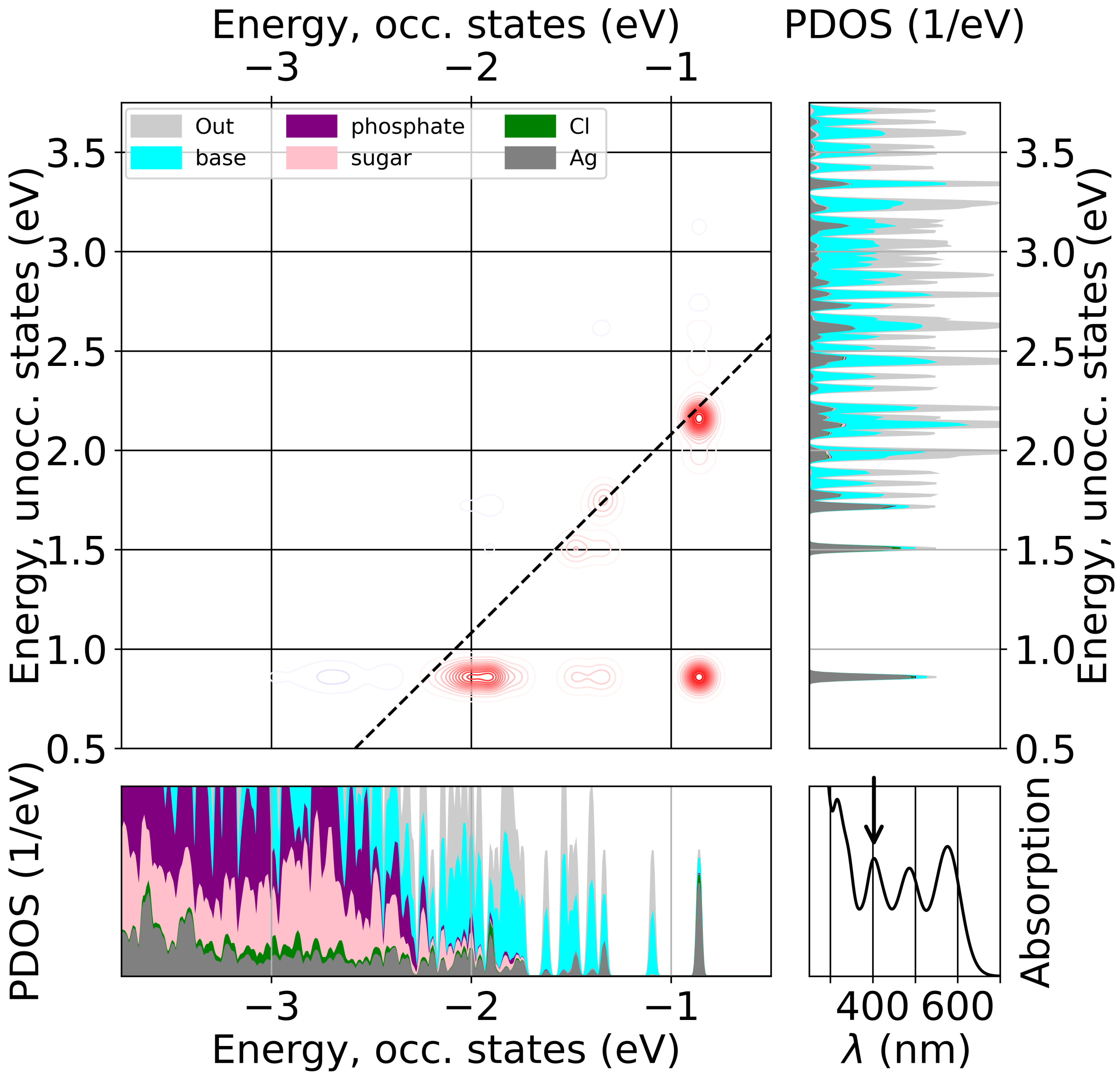

Figure S1: The Dipole Transition Contribution Map (DTCM) illustrates the higher energy absorption peak of sub-cluster **A** at 402 nm (indicated by the black arrow) in the absorption spectrum, computed using the GLLB-SC ground state with implicit solvent. The red spots in the top left panel highlight the combined transitions from silver-to-silver (HOMO-to-LUMO), silver-to-base, and base-to-silver. The bottom left panel depicts the projected densities of occupied states (PDOS), while the top right panel displays unoccupied states.

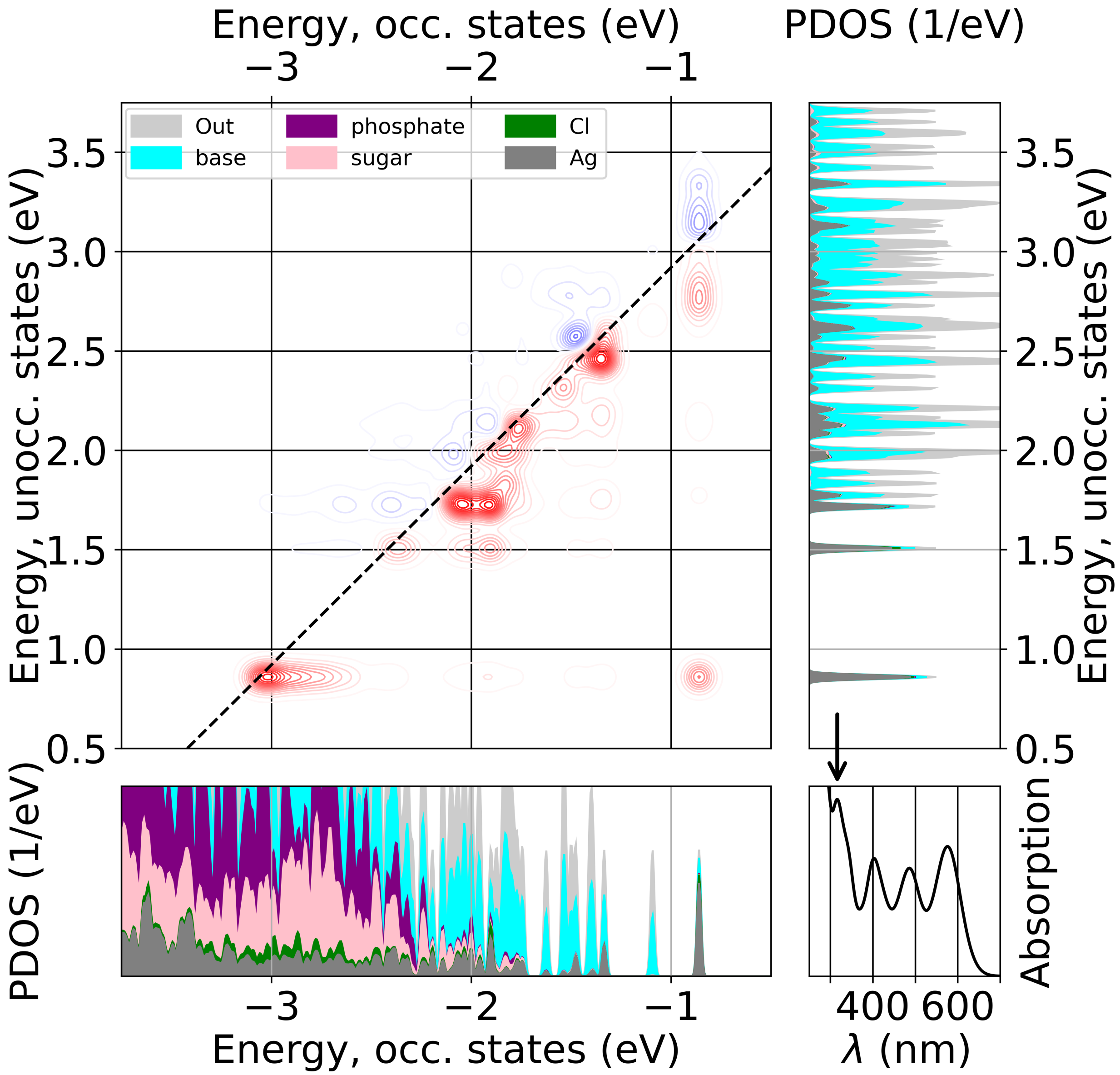

Figure S2: The Dipole Transition Contribution Map (DTCM) illustrates the higher energy absorption peak of sub-cluster **A** at 316 nm (indicated by the black arrow) in the absorption spectrum, computed using the GLLB-SC ground state with implicit solvent. The red spots in the top left panel highlight the collective transitions from silver-to-silver (HOMO-to-LUMO), silver-to-base, base-to-silver, base-to-base, and sugar-phosphate-to-silver. The bottom left panel depicts the projected densities of occupied states (PDOS), while the top right panel displays unoccupied states.
